# Faith-based organisations and HIV prevention in Africa: A review

**DOI:** 10.4102/phcfm.v5i1.464

**Published:** 2013-05-30

**Authors:** Rachel Mash, Robert Mash

**Affiliations:** 1 Division of Family Medicine and Primary Care, Stellenbosch University, South Africa

## Abstract

**Background:**

Faith-based organisations (FBOs) are potentially an important role-player in HIV prevention, but there has been little systematic study of their potential strengths and weaknesses in this area.

**Objectives:**

To identify the strengths and weaknesses of FBOs in terms of HIV prevention. The questions posed were, (1) ‘What is the influence of religion on sexual behaviour in Africa?’, and (2) ‘What are the factors that enable religion to have an influence on sexual behaviour?’.

**Method:**

A literature search of Medline, SABINET, Africa Wide NIPAD and Google Scholar was conducted.

**Results:**

The potential for Faith-based organisations to be important role-players in HIV prevention is undermined by the church’s difficulties with discussing sexuality, avoiding stigma, gender issues and acceptance of condoms. It appears that, in contrast with high-income countries, religiosity does not have an overall positive impact on risky sexual behaviour in Africa. Churches may, however, have a positive impact on alcohol use and its associated risky behaviour, as well as self-efficacy. The influence of the church on sexual behaviour may also be associated with the degree of social engagement and control within the church culture.

**Conclusion:**

Faith-based organisations have the potential to be an important role player in terms of HIV prevention. However, in order to be more effective, the church needs to take up the challenge of empowering young women, recognising the need for their sexually-active youth to use protection, reducing judgemental attitudes and changing the didactical methods used.

## Abstrait

Les organisations religieuses et la prévention du VIH en Afrique : Étude

**Présentation:**

Les organisations religieuses pourraient jouer un rôle important dans la prévention du VIH, mais peu d’études systématiques sont réalisées sur les forces et faiblesses observées dans ce domaine.

**Objectifs:**

Identifier les forces et faiblesses des organisations religieuses en termes de prévention du VIH. Les questions posées étaient les suivantes : (1) «Quelle est l’influence de la religion sur les comportements sexuels en Afrique», et (2) «Quels sont les facteurs permettant à la religion d’influencer le comportement sexuel?»

**Méthode:**

Une recherche documentaire a été réalisée dans Medline, SABINET, Africa Wide NIPAD et Google Scholar.

**Résultats:**

Le potentiel des organisations religieuses à jouer un rôle important dans la prévention du VIH est sapé par les difficultés rencontrées par l’église pour parler de sexualité, éviter la stigmatisation, intégrer les questions liées au genre et accepter les préservatifs. Par opposition avec les pays à revenu élevé, il semblerait que la religiosité n’ait pas d’impact positif général sur les comportements sexuels à risque en Afrique. Les églises peuvent cependant avoir un impact positif sur la consommation d’alcool et les comportements à risque qui y sont associés, ainsi que l’efficacité personnelle. L’influence de l’église sur le comportement sexuel pourrait également être associée au degré d’engagement social et de contrôle au sein de la culture de l’église.

**Conclusion:**

Les organisations religieuses pourraient jouer un rôle important en matière de prévention du VIH. Cependant, pour que celles-ci gagnent en efficacité, elles doivent s’attaquer au défi que représente l’autonomisation des jeunes femmes, reconnaître la nécessité que les jeunes sexuellement actifs se protègent, réduire les attitudes critiques et changer la didactique adoptée.

## Introduction

### Significance

This review highlights the important role of religion in terms of its impact on sexuality. Given the reach of Faith-based organisations (FBOs) in Africa, this review has some important findings with regard to HIV prevention: 

Religion is so overwhelmingly significant in… African values, attitudes, perspectives and decision-making frameworks that the inability to understand religion leads to an inability to understand people’s lives.^1^ 

### Aim of the article

Although African Faith-based organisations are recognised as being important role players in the provision of HIV care, there is less evidence of their engagement with HIV prevention programmes or in reducing risky sexual behaviour.^1,2,3^ The aim of this article is to review the literature in relation to the following three questions:

- What are the strengths and weaknesses of FBOs in terms of HIV prevention?
- What is the influence of FBOs on sexual behaviour in Africa?
- What are the factors that enable FBOs to have an influence on sexual behaviour? 

### Key word search

A search of Medline, SABINET, Africa Wide NIPAD and Google Scholar was conducted using the following key words: religion, faith-based, Christian, Islam, Muslim, HIV prevention, sexual activity, sexual behaviour, condoms, abstinence, Africa and Sub-Saharan Africa. The review included both medical and religious journals, but was limited to literature in English from 1990 onward. FBOs were defined as local religious congregations or religious non-profit organisations.^6^

## The strengths of Faith-based organisations in HIV prevention work 

Faith-based organisations are accessible throughout Africa and extend into the poorest informal settlements and the most remote villages.^3,4,5,6,7^ Interventions via FBOs are affordable since churches have existing infrastructure and personnel, and church members are often motivated by their faith and are willing to volunteer.^1,6^ FBOs also have a high level of acceptability, sometimes higher than government or foreign organisations, since they are part of the local culture.^1,3^ They are also known for their positive values such as justice, compassion and respect for human dignity.^3^ FBOs can have a positive role in facilitating behaviour change with a large constituency on a weekly basis, affording opportunities for information-sharing and teaching. Religions uphold the principles surrounding family, marriage and sexuality; promoting abstinence outside of marriage; and fidelity within marriage.^6,7^ FBOs, therefore, have the potential strength to be key role players in combating the HIV pandemic, but this potential is limited by several weaknesses.

## The weaknesses of Faith-based organisations in HIV prevention work

In the early days of HIV in Africa, the Church added to the stigma of those diagnosed with HIV. Many church leaders are not comfortable with speaking openly about sex and give inadequate attention to issues such as domestic violence or sexual coercion.^3,8^ The church has often ignored the needs of sexually-active adolescents and has enormous issues with the topics of gender and homosexuality.^6^ FBOs contributed to this stigma since many churches viewed HIV infection as being the consequence of immoral actions. Although the position of the church has changed slowly over the past twenty years, stigmatising attitudes continue at the local level.^3,6^ Churches have been criticised for patriarchal and hierarchical structures that promote gender inequality.^4,6,7^ The most highly-publicised negative role of the church is its attitude toward condoms; especially the Roman Catholic and Pentecostal Churches. As a Catholic priest in Kenya said: 

There is no place in our religion for the use of condoms, whether in the regulation of fertility or in the control of the diseases. And that is the teaching of Christ.^8^ 

Many Protestant churches also believe that teaching about condoms would encourage promiscuity. The condom debate tends to focus almost entirely on individual morality, sidelining issues such as socio-economic status, culture and gender relations.^8^ 

## The influence of Faith-based organisations on sexual behaviour 

Research conducted in high-income countries shows that religious faith plays a role in protecting young people from early sexual activity.^9,10,11, 12,13,14,15^ Religious youths were likely to have fewer sexual partners and to delay sexual initiation.^11,12,15,16,17^ However, studies also indicate that sexually-active religious youths were less likely to use protection due to the linking of condoms with sin.^13,14,15, 16,18^ Thus studies from high-income countries indicate that although religiosity may protect against initiating sexual activity, it may fail to protect against unsafe sex once youths are sexually active. There are a few studies on religiosity and sexual activity from Africa,^19,20,21,22^ and the eleven studies found are summarised in Table 1. 

**TABLE 1:**
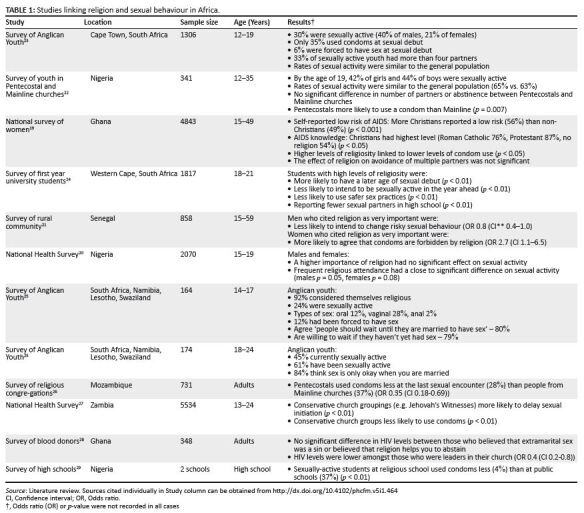
Studies linking religion and sexual behaviour in Africa.

### Sexual activity

Two studies reported a later age of sexual debut for religious youth, whereas three others showed no impact.^20,22,23,24,27^ Studies from South Africa and Nigeria found that sexual activity amongst church youth did not appear to differ substantially from that of the local community.^22,23,25,26^ As these studies are based in different cultural and theological groups, they suggest that the effect of religiosity on sexual behaviour might be less in Africa than in high-income countries. Only one study showed an impact of religiosity on numbers of partners whereas all the others showed no impact.^24^ Most of the studies showed a reduction in condom use and one study showed no impact.^27^

### Non-vaginal sex

In South Africa, church youth engage in oral and anal sex in order to maintain a technical virginity.^23,25^ Because the church often views these subjects as ‘taboo’ they are possibly exposing youth to risk through a lack of information.

### Gender

One study showed higher levels of sexual activity amongst religious girls, as compared with non-religious girls.^22^ Religious girls may also be at higher risk of HIV infection because they are more likely to see condoms as forbidden by their religion.^21^ They may also conform to religious teaching to be submissive, finding it harder to either refuse sex or negotiate for condom use. This finding contrasts with studies from high-income countries that show that religiosity can aid in reducing sexual activity amongst girls and could point to lower levels of self-efficacy amongst African women.^21^

### Sexual coercion

Rates of sexual coercion are high amongst church youth in some of these studies and this is an area of concern since few religious organisations deal with this issue openly.^23,25^

These findings suggest that religious people may be at higher risk of contracting HIV, since the protective effect of a raised age of sexual debut may be cancelled out by the negative impact of reduced condom use. The positive effect of religion which was noted in high-income countries appears to be weaker in Africa.

## How Faith-based organisations influence sexual behaviour 

Several factors determine the extent to which an individual’s behaviour is influenced by their religiosity. These include moral and religious teaching, socialisation within the group, level of attendance and commitment to the group.^27,30,31^

### Teaching on extramarital sex

Most religious groups are opposed to premarital and extramarital sex, but messaging varies between groups.^30,32^ In Zionist and Mainline Churches, the ‘approach seems to be that promiscuity is bad, but that abstinence is unrealistic, and that pre-marital sex with one partner is admissible’.^30^ In contrast, the Pentecostal churches give very clear directives against pre-marital sex:

‘The abasindiswa (Pentecostals) don’t have sex at all before marriage. But we amakholwa (Mainline) are more realistic, we know that we are human. So we do have our boyfriends, but just one at a time. Non-church goers have as many relationships as they want’.^30^ 

### Teaching on condoms

Although Pentecostal and Catholic churches take a stronger stance against condom usage than others, it appears that the official teaching of the Catholic Church is not always adhered to at grassroots level and some priests discuss condom use privately with parisioners.^31^ In contrast, the Pentecostal church appears to be strictly against condoms, at both official and grassroots levels. Some Pentecostal leaders described condoms as ‘satanic’ and ‘promoters of sin’.^32^ Amongst Mainline churches there is often more openness to the promotion of condoms through the ABC (Abstain, Be faithful, Condomise) message. However, many leaders feel that these are conflicting messages:

I find it difficult to tell my members to use Chishango (condoms) should they fail to abstain. I tell someone that doing this is sinning. I have disseminated two different messages at once.^31^ 

### Methods of teaching

Current moral teaching does not appear to be effective in leading to behaviour change and churches often address issues of sexuality in didactic and judgemental ways.^6^ Young people are told to stay away from sex, but are not empowered with the skills to be able to do so. Changing values and behaviour requires interaction, discussion and clarification.^[Bibr CIT0033]^ In order to adopt and internalise new values, interactive dialogical methods of teaching are likely to be more effective. Through participatory educational methods, young people can apply their spiritual values to the area of sexuality as well as develop a critical consciousness around their gender roles that protects them better from unsafe sex.^34^

### Attitudes to alcohol

Religious groups tend to have more conservative attitudes to alcohol, which may lead to a reduction in levels of unsafe sexual behaviour (as this is often seen to occur under the influence of alcohol).^9,35^ 

### Building self-efficacy 

Religion helps people in their search for meaning and associations have been found between self-efficacy and religiosity.^9,12^ Self-efficacy can be increased as young people are affirmed and given opportunities for leadership.^36^

### Social groups

The more deeply involved religion is in daily life, the greater its ability to influence behaviour.^3,30^ In South Africa, Pentecostal youth meet about five times a week and this group has a strong influence on their attitudes and behaviour, helping them to abide by stricter rules. In contrast, few of the Mainline churches have youth meetings more than once a week:

Given the muted religious experience of Mainline Christianity, the absence of youth groups or choirs (socialisation) and the lack of specific teaching on sex-related matters (indoctrination) the influence of these churches on sexual praxis is limited.^30^ 

Social capital can be increased as young people become more involved in the church through positive peer pressure and by building relationships with adults who are positive role models.^31,37,38^

### Social control

Conservative denominations also exercise control over young people’s sexual behaviour. In Zambia, the ‘New Mission’ churches (e.g. Jehovah’s Witness, Seventh Day Adventist) had a greater degree of social control than other denominations as those found guilty of premarital sex were removed from membership. Other denominations are more flexible, with doctrines based on repentance and forgiveness.^27,32^

### Level of commitment

Sexual behaviour is also impacted by a person’s level of commitment to their religion.^27,31,39^ With greater levels of commitment, an individual will receive more frequent religious messages and will socialise with peers with similar attitudes.^40^ Fatusi’s study in Nigeria found that increased attendance at church increased sexual activity, whereas those who attached a higher level of importance to religion had lower rates of sexual activity.^20^ This could indicate that, in certain communities, attending church is one of the ways to meet sexual partners. Myint’s study^41^ also indicated that HIV-positive people may attend church more frequently as a source of support.

## Limitations of the review

This was an extensive review of the literature as part of a doctoral thesis, however the methodological rigour of a systematic review on one specific research question was not followed. The literature found was very limited and, as a result of its paucity, it was necessary to include studies which were not methodologically of a high standard. For example, details of the methods and outcome measures were often not well defined. Although the literature is reviewed in terms of an African perspective, the huge variety of contexts, cultures and religions within Africa make it difficult to generalise. The findings of this review should therefore be seen as preliminary and tentative. Nevertheless, the review summarises the available literature in an aspect of HIV prevention that is often overlooked from a health perspective. There is a need for further research to explore the suggested trends and associations. The majority of the literature was from the Christian tradition and very little was found from other religious traditions. 

## Conclusions

In Sub-Saharan Africa, given the church’s accessibility, affordability and acceptability, FBOs are potentially-important role players in HIV prevention. However, this potential is undermined by the church’s difficulties with discussing sexuality, avoiding the stigma of HIV, promoting gender equality and accepting the use of condoms.

In contrast with high-income countries, preliminary findings suggest that religiosity does not have a positive impact on sexual behaviour in Africa, apart from on the most committed members. In general, church-based youth may be at higher risk than the general population since sexual activity is similar, but they are less likely to use protection. Young women in particular appear to be at risk. The church needs to take up the challenge of empowering young women as well as recognising the need for protection of their sexually-active youth.

Faith-based organisations influence sexual behaviour through their moral and religious teaching, but differ significantly between denominations in the content of the teaching and usually do not engage with effective teaching methods. They may, however, have a positive impact on alcohol use, self-efficacy and peer pressure through social engagement and control. The level of an individual’s commitment is also associated with the effect on sexual behaviour.
